# Biological Evaluation of Some Amino Acids Esters: A Study on Antimicrobial, Antibiofilm, and Molecular Docking

**DOI:** 10.1021/acsomega.5c13628

**Published:** 2026-04-04

**Authors:** Tuğçe Deniz Karaca

**Affiliations:** Department of Medical Services and Techniques, Health Services Vocational School 37511 Gazi University, 06830 Ankara, Gölbaşı, Türkiye

## Abstract

The increasing emergence of multidrug-resistant microorganisms poses one of the most significant challenges in the clinical management of infectious diseases. This situation has made it necessary to research new, effective antimicrobial agents. The amino acid skeleton is vital for biological activities. Therefore, amino acid-based derivatives, which constitute a group of compounds with various biological activities, have found many applications in the medical field due to their high levels of biocompatibility and biodegradability. In this study, the antimicrobial and antibiofilm effects of some water-soluble amino acid alkyl esters (glycine benzyl ester, glycine *t*-butyl ester, glycine ethyl ester, l-phenylalanine benzyl ester, l-phenylalanine *t*-butyl ester, and l-phenylalanine ethyl ester) were investigated against the causative agents of hospital-acquired infections and strong biofilm formers, *Escherichia coli* OR651248 and *Staphylococcus aureus* ATCC 25923. Minimum inhibitory concentrations (MICs) and minimum bactericidal concentrations (MBCs) were determined by the broth dilution method. The Kirby–Bauer disk diffusion method was used to assess zones of inhibition (ZOIs), and biofilm formation was established by using the crystal violet binding assay. Amino acid esters exhibited notable antimicrobial and antibiofilm activities, suggesting their potential as alternative or adjunct antimicrobial candidates, and l-phenylalanine benzyl ester emerged as the most antimicrobial ester among all esters and showed the lowest MIC (1.56 mg/mL) and MBC (3.12 mg/mL) values against both *Escherichia coli* OR651248 and *Staphylococcus aureus* ATCC 25923 strains. Additionally, it was determined that glycine esters had a more effective biofilm inhibitory effect compared with l-phenylalanine esters. Also, molecular docking studies, ADME properties, and drug-likeness were also reported to support experimental findings, and these molecules were evaluated for their potential as inhibitors against Gram-negative *Escherichia coli* and Gram-positive *Staphylococcus aureus* using the antibacterial receptor (PDB:1HNJ). The theoretical docking simulations were performed with Autodock Vina software. These new findings, specific to this study, are thought to provide valuable data on the antimicrobial and antibiofilm effects of amino acid esters and contribute to the development of new synthesizable drug studies.

## Graphical abstract

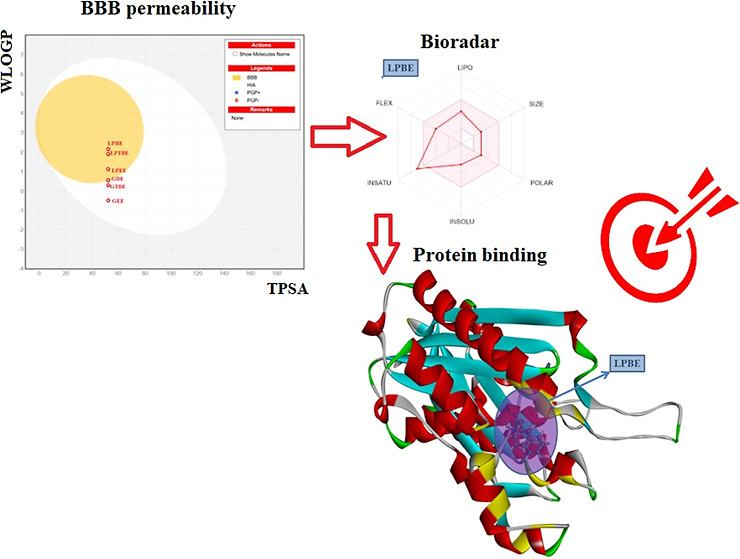

## 1 Introduction

The rapid increase in multidrug-resistant bacteria poses a serious threat to society. Antibiotic resistance in bacteria occurs through different resistance mechanisms and is mainly caused by overuse and misuse of antibiotics.^1^ Also, biofilm formation is an important virulence factor that increases the resistance of microbes to antimicrobials, and biofilm-induced infections have become an extremely important threat to humanity because biofilms easily adapt to changing environmental conditions and therefore resist traditional antibiotics.^2^

In this sense, *Escherichia coli* (*E. coli*) is a well-established microorganism that plays an important role in the human microbiome. However, some strains can become pathogenic, causing infections both in the intestinal tract and in other areas of the human body, where they can form biofilms. Also, *Staphylococcus aureus* (*S. aureus*) is a drug-resistant pathogen that can cause a variety of infections, including serious endocarditis, osteomyelitis, pneumonia, and other invasive diseases. In addition to abundant secreted virulence factors, biofilm formation is an important feature that promotes the development of drug resistance against *S. aureus*.^3,4^ Recently, in the study conducted with 107.053 culture-positive cases of drug-resistant pathogens, the highest number of infections was recorded for *E. coli*, followed by *S. aureus*.^5^ Therefore, the demand for new drugs and alternative therapeutic agents is increasing, and there is an inevitable need for studies that include all kinds of molecules that will contribute to the pharmacological treatment of patients with infections caused by resistant microorganisms.

In this context, amino acids and their derivatives with diverse biological activities constitute an important group of drug or drug candidate products due to their high levels of biocompatibility and biodegradability, thus offering an attractive range of biological products with great potential for drug development. For this reason, it has found various application areas such as antibiofilm agents, drug adjuvants, drug solubility enhancers, drug modifications, and drug excipients.^6–8^

Glycine is the most common and the simplest nonessential amino acid in humans and quite effective in improving the health of humans and animals and promoting growth. However, the effects of glycine on bacteria are remarkable, and an excess of glycine inhibits the growth of many bacteria. High glycine concentrations cause bacteriolysis and morphological changes in different bacteria. The main mechanism of glycine’s antimicrobial activity is the inhibition of cell wall synthesis.^9–12^ Maculla and Cowles demonstrated that bacterial cells were lysed by adding glycine to broth cultures.^13^ The antimicrobial effectiveness of glycine against *H. pylori* was also investigated, and significant results were obtained.^14^ There is also a recent study using glycine in *E. coli*.^15^ Moreover, studies conducted with some different glycine derivatives are noteworthy. l-α-Ethynylglycine is also stated to exhibit activity against Gram-positive bacteria^16^ and Thornberry et al. determined 3-halovinylglycines as a new class of potent irreversible inactivators of Alr from *E. coli*.^17^

Additionally, some studies have reported different results regarding the effect of the aromatic group in amino acids on the antibacterial activity. The presence of aromatic rings appears to increase the antimicrobial activity. Phenylalanine is also an amino acid containing an aromatic benzene ring, and it is converted to tyrosine and other amino acids, which are used in biosynthesis and therefore studies with phenylalanine derivatives are important.^18,19^ Also, a number of cationic molecules derived from l-phenylalanine have been evaluated for their antibacterial activity and found to be effective against both Gram-positive and Gram-negative bacteria.^20^ The antibiofilm activity of phenylalanine with a 9-fluorenylmethyloxycarbonyl group was clinically evaluated against bacteria, and it was found that it prevented biofilm formation in both *S. aureus* and *P. aeruginosa*.^21^ Moreover, there are antimicrobial studies on some metal complexes of both glycine and phenylalanine.^22^

Especially in the literature, it is emphasized that esters of amino acids show good antimicrobial activity against pathogenic microorganisms. Concurrent studies suggest that materials composed of ester bonds between amino acids and polysaccharides are useful for targeted drug delivery, bioimaging, or surface functionalization.^18,19^ Also, esters are reported to be a more promising choice because they can be easily hydrolyzed both in vivo and in vitro.^20^ However, there are very few studies in the literature on amino acid esters derivatives, which have been determined to have antibacterial studies.^21–26^ There has been no research to date investigating the antimicrobial and antibiofilm effects of glycine and phenylalanine alkyl ester forms of the amino acids on *S. aureus* and *E. coli* strains.

Therefore, further research on ester derivatives of amino acids is needed, and, in this study, antimicrobial and antibiofilm effects of some glycine and l-phenylalanine alkyl (R = benzyl, *t*-butyl, ethyl) esters against *E. coli* OR651248 and *S. aureus* ATCC 25923. Also conducted molecular docking studies of the compounds. The results of the study are noteworthy and will contribute to further research on amino acid-ester compounds. They will also guide drug development research by contributing to the development of new strategies using synthesizable ester-based molecules.

## 2 Materials and Methods

### 2.1 Glycine and l-Phenylalanine Esters and Their Preparations

Water-soluble glycine and l-phenylalanine esters presented in Table 1 were purchased from Sigma (>99.0% purity) and used in this study. They were dissolved in sterile PBS buffer solution (pH 7) and vortexed for 2 min to obtain stock ester solutions. The solutions were sterilized under UV light for 1 h to avoid potential compound loss during membrane filtration, and the chemical stability of the esters under UV exposure was taken into consideration.

**Table 1:**
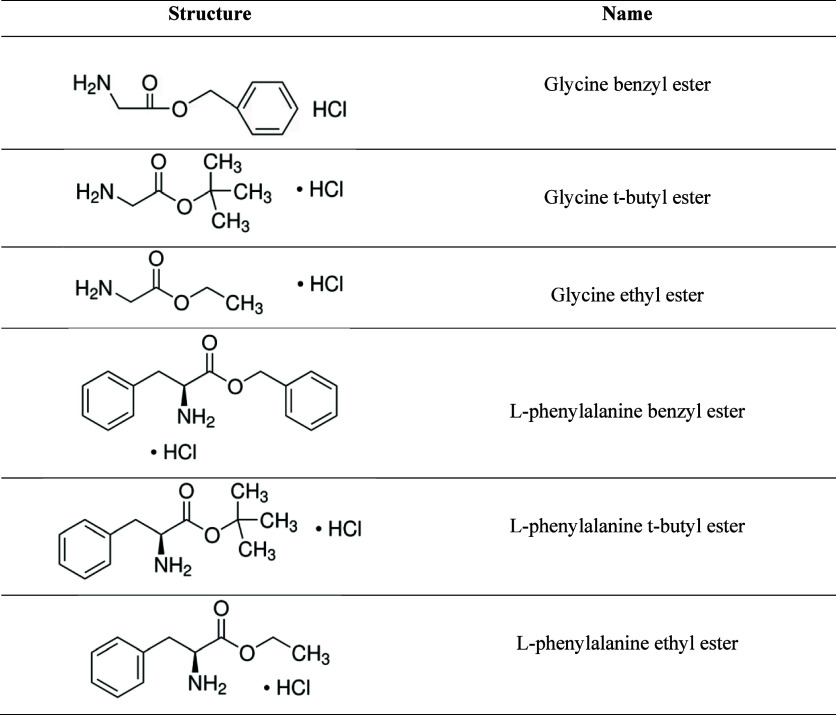
Structure and Names of Glycine and l-Phenylalanine Esters Studied

### 2.2 Microbial Strains

In this study, *S. aureus* ATCC 25923 and *E. coli* OR651248 were used.^27^ To obtain single colonies, both strains were inoculated onto Brain Heart Infusion (BHI) agar media from stock cultures using the streak plate method and were then incubated at 37 °C for 24 h. The resulting cultures were stored at 4 °C and underwent monthly passaging for use in future experiments.

### 2.3 Determination of the Antimicrobial Effect

To determine antimicrobial effects, broth dilution and disc diffusion methods were used.^28,29^ Microorganisms were grown and harvested, as described by Sahal et al.^27^ Briefly, single colonies belonging to microbial strains were cultured in 10 mL of BHI broth media at 37 °C for 24 h to establish precultures. Subsequently, 1.5 mL of the precultures was transferred into 30 mL of BHI broth and then incubated at 37 °C for another 24 h to develop the main cultures. The main cultures were harvested through centrifugation, performing three rounds at 3220*g* for 10 min at 5 °C (Eppendorf 5810R centrifuge with an Eppendorf Swing-bucket rotor A-4–62 in Hamburg, Germany). A potassium phosphate buffer (pH 7) was used as a washing buffer. Microbial cell densities were adjusted to the MacFarland (MF) 2 turbidity standard for use in subsequent experiments.

#### 2.3.1 Broth Dilution Method

In each well of 96-well plates, 100 μL of a stock ester solution (at a concentration of 400 mg/mL) was added to 100 μL of BHI broth. 2-fold serial dilutions were then carried out to establish a range of ester concentrations, covering values from 0.39 to 200 mg/mL (Ester/BHI). Following this, 15 μL of microbial cells, which had been adjusted to the MF 2 turbidity standard in a potassium phosphate buffer (pH 7) (as previously described), were inoculated into the wells. The 96-well plates were incubated at 37 °C for 24 h. After the incubation, visual examinations were conducted to detect growth, and the minimum concentration at which no growth was observed was determined as the minimum inhibitory concentration (MIC). Subsequently, 10 μL samples were collected from the 96-well plates and transferred to BHI agar plates to observe growth in a medium without ester inclusion. The minimum concentration of esters in the wells, at which no growth was observed on the BHI agar plate, was identified as the minimum bactericidal concentration (MBC). All experiments were conducted in triplicate at a minimum, and median values were reported.

#### 2.3.2 Disc Diffusion Method

20 μL of each stock ester solution (at a concentration of 100 mg/mL) was applied onto sterile discs (Whatman paper, Z741310, microbial assays). As a negative control, a disc loaded with 20 μL of sterile distilled water was included. These discs were then positioned onto BHI agar media that had been previously inoculated with 100 μL of microbial cells adjusted to the MF 2 turbidity standard in a potassium phosphate buffer (pH 7) (as previously described). The BHI agar plates were subsequently incubated at 37 °C for 24 h. Following incubation, the diameters of the Zones of Inhibitions (ZOIs) were measured using a ruler, and the measurements were recorded.

### 2.4 Determination of Biofilm Inhibition

Biofilm inhibition resulting from sub-MIC values of the tested esters was assessed via the modified crystal violet binding assay described by Sahal et al.^30,31^ In summary, solutions of amino acid esters at 0.25 times the MIC for each strain were prepared in 96-well plates. 15 μL of microbial cells, adjusted to the MF 2 turbidity standard in potassium phosphate buffer (pH 7), were added to the wells. The 96-well plates were then incubated at 37 °C for 24 h. After incubation, the wells were washed three times with sterile distilled water and stained with crystal violet for 30 m. Following additional rinsing with sterile distilled water, the bound crystal violet, indicating biofilm formation, was dissolved using a 99.9% ethanol (Merck) solution. The solubilized bound crystal violet was measured at 560 nm using a microplate spectrophotometer (BIO-TEK μQuant, BIO-TEK Instruments, Inc.) reader. Wells without any microbial inoculation were set as a negative control, while wells without any amino acid ester inclusion were set as a positive control, with their biofilm formation set at 100%. To calculate the reduction (%) in biofilm formation compared with the positive control, the following formula was applied:

% Decrease = [(Absorbance Control at 560 nm) – (Absorbance Treatment at 560 nm)]/(Absorbance Control at 560 nm) × 100 %.

All experiments were performed in triplicate with bacterial cells cultured separately, and the results show the mean values of percentage inhibitions.

### 2.5 Statistical Analysis

The Shapiro–Wilk test was used to assess the normality of the data. If the data were not normally distributed, the Kruskal–Wallis test was applied for comparisons of more than two groups to evaluate especially ZOI and biofilm inhibition results. A *p*-value of less than 0.05 was considered statistically significant. Statistical analysis was performed using SPSS version 23 (IBM Corp, New York, USA).

In silico docking analyses were performed using the AutoDock Vina program.^32^ The molecular structures of esters were optimized in their ground state and gas-phase configurations using Density Functional Theory (DFT) with the B3LYP functional and 6–311++G­(d,p) basis set, implemented in the Gaussian 09W software package.^33^ The optimized structures were visualized in three dimensions using the GaussView program,^34^ without imposing any symmetry constraints. (The esters are shown as follows: Glycine Benzyl Ester: GBE, Glycine *t*-butyl ester: GTBE, Glycine ethyl ester: GEE, l-phenylalanine benzyl ester: LPBE, l-phenylalanine *t*-butyl ester: LPTBE, and l-phenylalanine ethyl ester: LPEE)

## 3 Results

### 3.1 Antimicrobial Effects

When examining the MICs and MBCs of glycine and l-phenylalanine esters against *E. coli* OR651248 and *S. aureus* ATCC 25923 for their antimicrobial effects, it was observed that while both strains were equally susceptible to glycine esters, *E. coli* was more susceptible to l-phenylalanine esters. This is evidenced by the lower MIC and MBC values for *E. coli* OR651248 compared to those of *S. aureus* ATCC 25923 (Table 2). On the other hand, l-phenylalanine benzyl ester emerged as the most antimicrobial ester among all the tested l-phenylalanine esters, exhibiting the lowest MIC and MBC values against both *E. coli* OR651248 and *S. aureus* ATCC 25923 strains (Table 2). In contrast, glycine ethyl ester was the least effective ester, with the highest MIC and MBC values observed against both the *E. coli* OR651248 and *S. aureus* ATCC 25923 strains (Table 2).

**Table 2:** MICs (MICs; mg/mL; Median Values, *n* = 3), and MBCs (MBCs; Mg/mL; Median Values, *n* = 3) Against *E. coli* OR651248 and *S. aureus* ATCC 25923 Strains

	E. coli OR651248		S. aureus ATCC 25923	
	**MIC** (mg/mL)	**MBC** (mg/mL)	**MIC** (mg/mL)	**MBC** (mg/mL)
Glycine benzyl ester	12.5	25	12.5	25
Glycine t-butyl ester	25	50	25.0	50
Glycine ethyl ester	200	>200	200	>200
l-phenylalanine benzyl ester	1.56	3.125	3.1	3.13
l-phenylalanine t-butyl ester	6.25	6.25	12.5	12.5
l-phenylalanine ethyl ester	12.5	50	50.0	50
Penicillin–Streptomycin	250	500	15.6	15.6

No inhibition zone was observed for certain compounds under the tested conditions. Based on the disc diffusion test results, glycine *t*-butyl ester and glycine ethyl ester did not produce any zones of inhibition against either the *E. coli* OR651248 or *S. aureus* ATCC 25923 strains (Figures 1 and 2). In contrast, the l-phenylalanine benzyl ester exhibited the largest zone of inhibition diameters, measuring 12.5 mm for *E. coli* OR651248 and 11 mm for *S. aureus* ATCC 25923, respectively (Figures 1 and 2). Additionally, l-phenylalanine ethyl ester only displayed a ZOI against *E. coli* OR651248.

**Figure 1:**
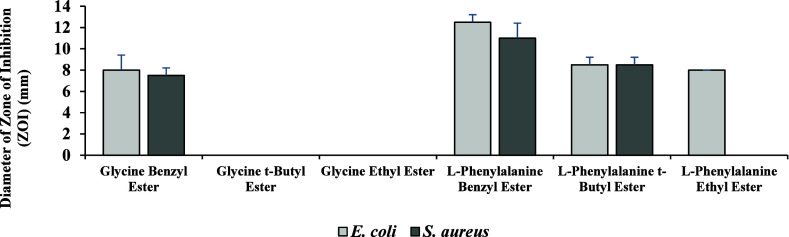
Diameter of ZOIs of *E. coli* OR651248 and *S. aureus* ATCC25923 strains against glycine and l-phenylalanine esters.

**Figure 2:**
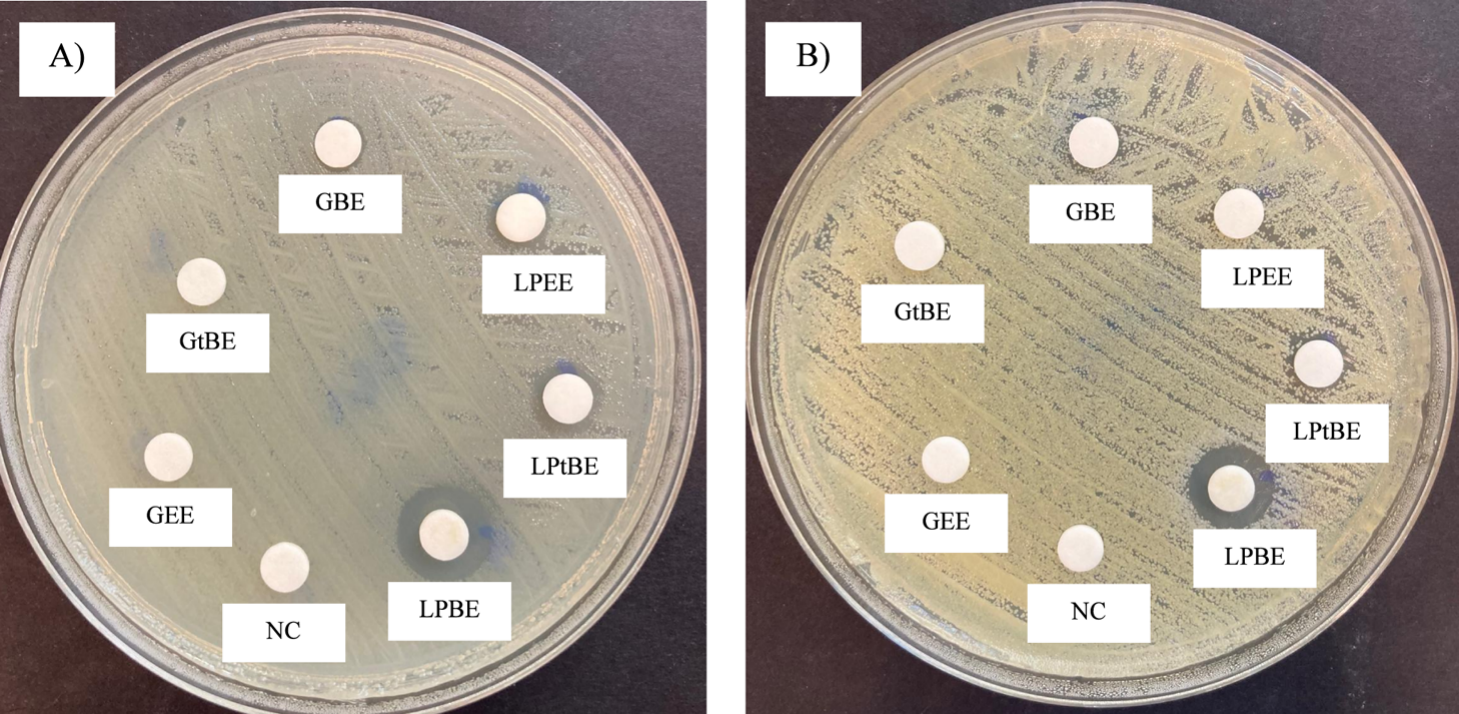
Image displays zones of inhibition (ZOIs) produced by glycine and l-phenylalanine esters against (A) *E. coli* OR651248 and (B) *S. aureus* ATCC 25923 (NC: Negative Control).

According to the Shapiro–Wilk test results, the data for ZOIs were not normally distributed for both *E. coli* OR651248 (*p* = 0.003) and *S. aureus* ATCC 25923 (*p* < 0.001) strains. Therefore, Kruskal–Wallis was applied to compare the significant differences between ZOIs caused by the tested amino acid esters, and a significant difference was found between different amino acid esters for both *E. coli* OR651248 (*p* = 0.009) and *S. aureus* ATCC 25923 (*p* = 0.005) strains.

### 3.2 Biofilm Inhibitions

In this part of this study, all of the glycine and l-phenylalanine esters tested exhibited biofilm inhibition against both *E. coli* OR651248 and *S. aureus* ATCC 25923. Figure 3 demonstrates that while 0.25 MIC l-phenylalanine esters caused 20% to 22% inhibition of biofilm formation in *E. coli* OR651248 and 27% to 43% in *S. aureus* ATCC 25923, glycine esters caused more than 50% biofilm inhibition against each tested strain (Figures 3 and 4).

**Figure 3:**
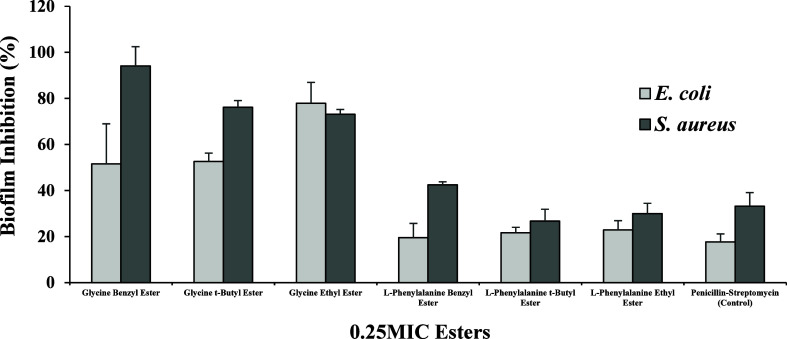
Biofilm inhibitory effect of 0.25MIC of glycine and l-phenylalanine esters against *E. coli* OR651248 and *S. aureus* ATCC 25923 strains.

**Figure 4:**
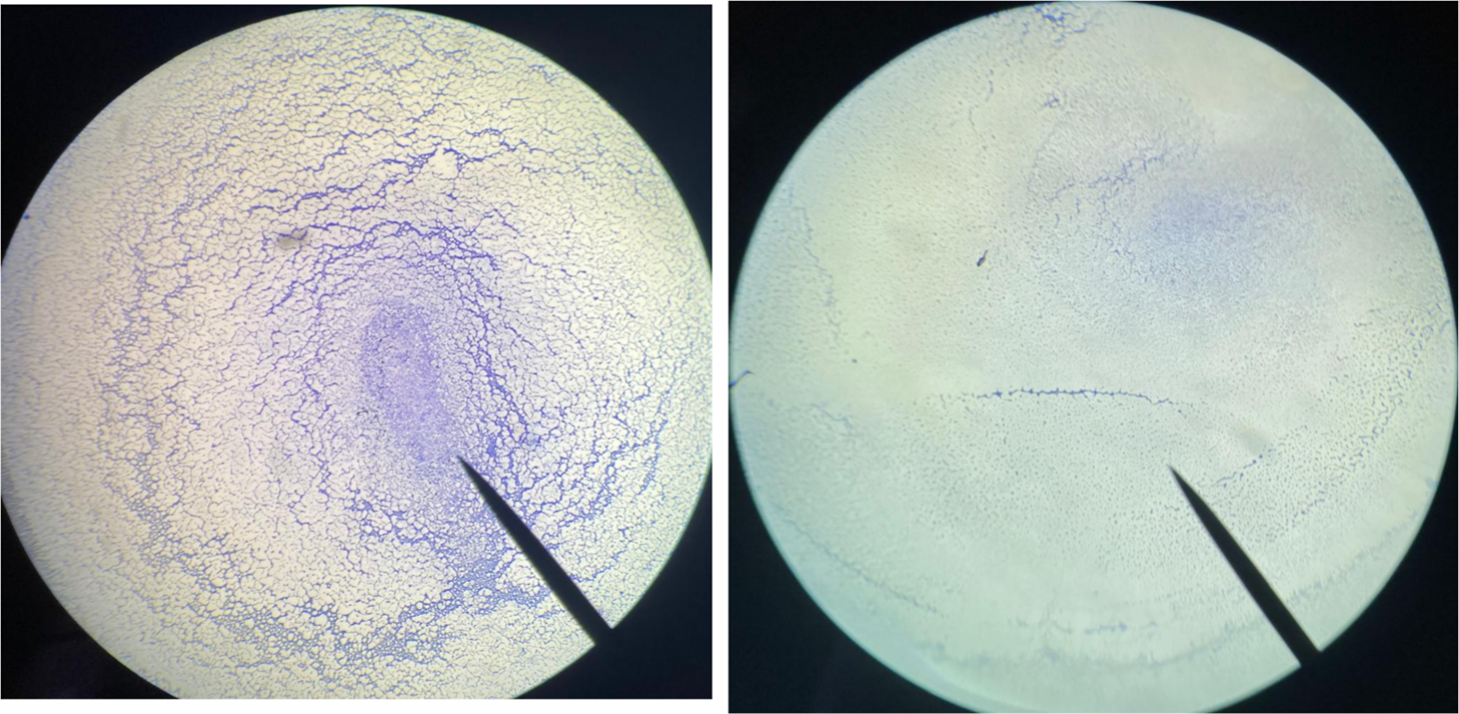
Light microscopy images of *S. aureus* ATCC 25923 showing (A) untreated sample and (B) sample treated with 0.25 MIC of glycine benzyl ester. The images were captured using a brightfield light microscope at 4× magnification (LEICA DM500). The violet color indicates biofilm formation.

According to the Shapiro–Wilk test results, the data for percentage biofilm inhibition was not normally distributed (*p* = 0.003) for both *E. coli* OR651248 and *S. aureus* ATCC 25923 (*p* = 0.009) strains. According to the Kruskal–Wallis test, a significant difference was found between different amino acid esters for both *E. coli* OR651248 (*p* = 0.007) and *S. aureus* ATCC 25923 (*p* = 0.005) strains. The biofilm inhibition caused by glycine ethyl ester against *E. coli* OR651248 was higher than that caused by all of the other tested esters (Figures 3 and 4). In addition, the biofilm inhibitory effect of all glycine esters was significantly higher than the effect caused by all l-phenylalanine esters (*p* < 0.05) (Figure 3). The highest biofilm inhibitory effects were observed with glycine benzyl ester, glycine *t*-butyl ester, and glycine ethyl ester against *S. aureus* ATCC 25923, with 94%, 76%, and 73% biofilm inhibitions, respectively (Figures 3 and 4), which were significantly higher than the effects caused by l-phenylalanine esters (*p* < 0.05) (Figures 3 and 4).

#### 3.2.1 Molecular Docking Analysis

Molecular docking is a popular method that helps us characterize how small molecules behave at the binding sites of target proteins and better understand fundamental biological processes by simulating the interaction between a small molecule and a protein at the atomic level. Understanding binding mechanisms, locating possible therapeutic options, and evaluating the potency and specificity of ligand–receptor interactions all depend on this technique. Researchers can assess the spatial fit of ligands within a target’s active site and determine binding affinities by modeling the docking process. The binding energy, a measure of the strength of the contact is quantified by molecular docking using scoring functions. The findings help to guide the design and optimization of bioactive chemicals by offering insightful information about molecular recognition processes. The accuracy and efficiency of these analyses have been improved by developments in docking algorithms and software, such as AutoDock Vina and Glide, making them essential instruments in contemporary molecular biology and drug discovery research.^35–37^ The Autodock Vina program was used to finish the molecular docking calculations.^32^ The theoretically derived optimizations were utilized to create the PDB structures for the ligand molecules. All ligand-related processes, including converting the ligands into PDB and PDBQT formats, were carried out using the Discovery Studio Visualizer 4.0 (DSV 4.0) application.^38^ The three-dimensional PDB structure of this receptor (their potential as inhibitors against Gram-negative *E. coli* (OR651248) and Gram-positive *S. aureus* (ATCC 25923) using the antibacterial receptor (PDB:1HNJ)) was made available by the RCSB Protein Data Bank (PDB).^39^ Discovery Studio Visualizer 4.0 (DSV 4.0) was also used to remove heteroatoms that were discovered in the target protein.^38^ The processed data were again recorded in PDB and PDBQT formats after the addition of polar hydrogen atoms and the corresponding charges. To increase the precision of the docking study, the target protein’s essential residues and active sites were identified as follows: GLY209, MET207, GLU171, ILE155, ARG151, THR37, ARG36, TRP32, THR28, ASP21, VAL16, ASN247, PHE213, and ASN210 for PDB:1HNJ. These active zones were taken into consideration when creating the grid parameters, which are as follows: 54 × 40 × 56 Å^3^ *x*, *y*, *z* dimensions, 0.375 Å space, and 29.971, 6.57, 30.364 *x*, *y*, *z* centers for PDB: 1HNJ. Table 3 lists interactions with related proteins as well as the molecular docking data for the esters. Furthermore, Table 3 displays the values of *K*_*i*_, or the inhibition constant, which is determined using the standard hydrogen bond numbers and binding energies created during molecular–protein interactions. Ligand + related protein inhibition values were computed using the *K*_i_ = exp­(-G/*RT*) equation (“G-binding energy, R-gas constant = 1.9872036 × 10–3 kcal/mol, and *T*-temperature = 298.15 K”). As can be seen from Table 3, the lowest binding energy between the l-phenylalanine benzyl ester molecule and the PDB:1HNJ protein is −8.8 kcal/mol, and the structure contains two conventional hydrogen bonds. The localization of the molecule to the active site of the protein is shown in Figure 5 and the 3D (a) and 2D forms of docking (with b-bond lengths) are given in Figure 6. Three-dimensional docking results of the interactions between glycine benzyl ester, glycine *t*-butyl ester, l-phenylalanine ethyl ester, and l-phenylalanine *t*-butyl ester molecules and 1HNJ protein were also shown in Figures S1–S4. Table 3 summarizes the docking scores, including binding energies (Δ*G*), the number of hydrogen bonds, and inhibition constants (*K*_i_). Among the ligands, l-phenylalanine benzyl ester demonstrated the strongest binding affinity to the 1HNJ receptor, with an Δ*G* of −8.8 kcal/mol and two hydrogen bonds, corresponding to a *K*_i_ of 0.97579 μM. l-phenylalanine ethyl ester and l-phenylalanine *t*-butyl ester also exhibited significant binding affinities with Δ*G* values of −8.7 kcal/mol and one hydrogen bond each. In contrast, glycine *t*-butyl ester and glycine ethyl ester showed the weakest interaction, with an Δ*G* of −6.9 kcal/mol.

**Table 3:** Molecular Docking Scores of the Esters

Compounds	PDB1HNJ
Glycine benzyl ester	*E* = −8.2 kcal/mol
	The number of hydrogen bonding = 1
	*K*_i_ = 0.97579 μM
Glycine *t*-butyl ester	*E* = −6.9 kcal/mol
	The number of hydrogen bonding = 1
	*K*_i_ = 8.75514 μM
Glycine ethyl ester	*E* = −6.9 kcal/mol
	The number of hydrogen bonding = 1
	*K*_i_ = 8.75514 μM
l-Phenylalanine benzyl ester	*E* = −8.8 kcal/mol
	The number of hydrogen bonding = 2
	*K*_i_ = 0.97579 μM
l-Phenylalanine *t*-butyl ester	*E* = −8.7 kcal/mol
	The number of hydrogen bonding = 1
	*K*_i_ = 0.419618 μM
l-Phenylalanine ethyl ester	*E* = −8.7 kcal/mol
	The number of Hydrogen Bonding = 1
	*K*_i_ = 0.419618 μM

**Figure 5:**
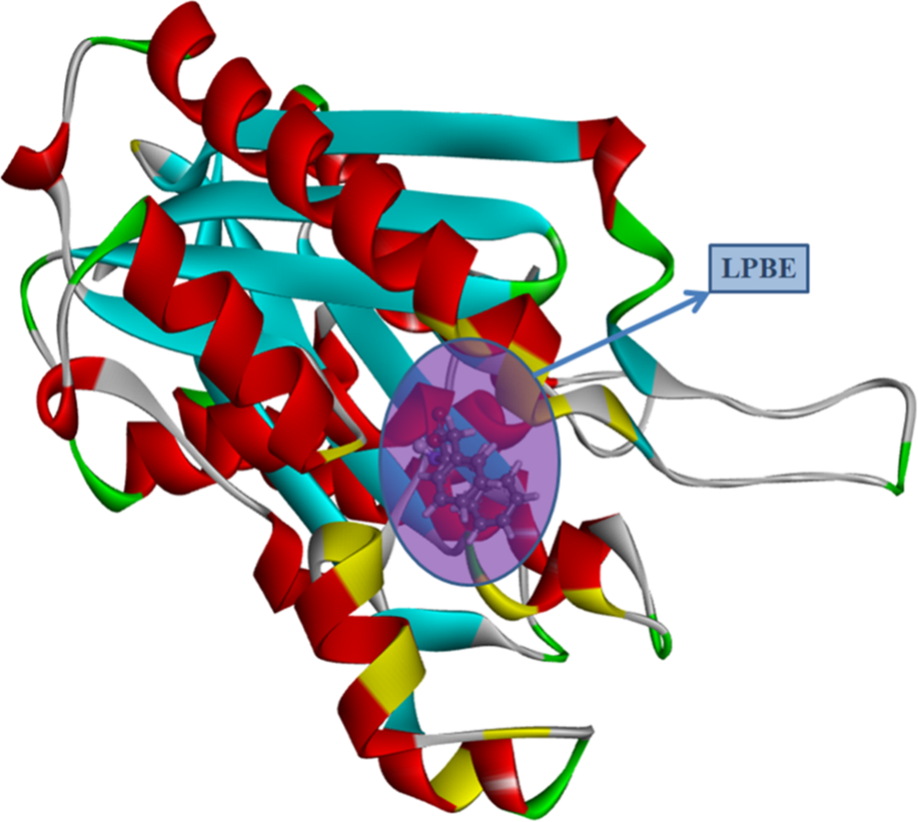
Localization of the molecule (l-phenylalanine benzyl ester) to the active sites of the protein.

**Figure 6:**
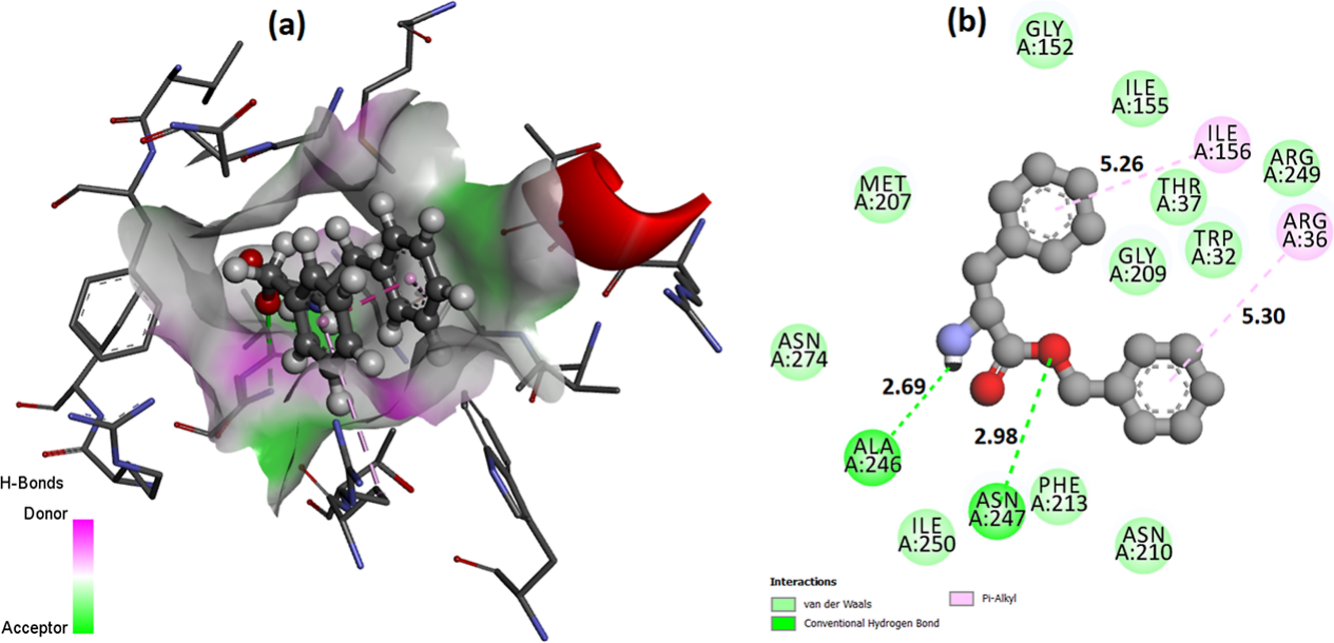
3D (a) and 2D (b) molecular docking results for compound l-phenylalanine benzyl ester and PDB: 1HNJ.

#### 3.2.2 ADME Analysis

The pharmacokinetic and physicochemical profiles of amino acid esters were evaluated using the SwissADME platform to predict their drug-likeness and metabolic fate (Table S1). The WLOGP vs TPSA (boiled egg) graph, used to estimate the gastrointestinal absorption and brain penetration of the studied amino acid esters, is given in Figure 7. In the next step, bioavailability radars of the amino acid esters were created, as shown in Figure 8 by considering parameters such as lipophilicity, size, polarity, solubility, flexibility, and saturation. When a molecule’s radar plot fully satisfies a set of physicochemical criteria for each description, it is indicated by a pink area as being drug-like.^40^

**Figure 7:**
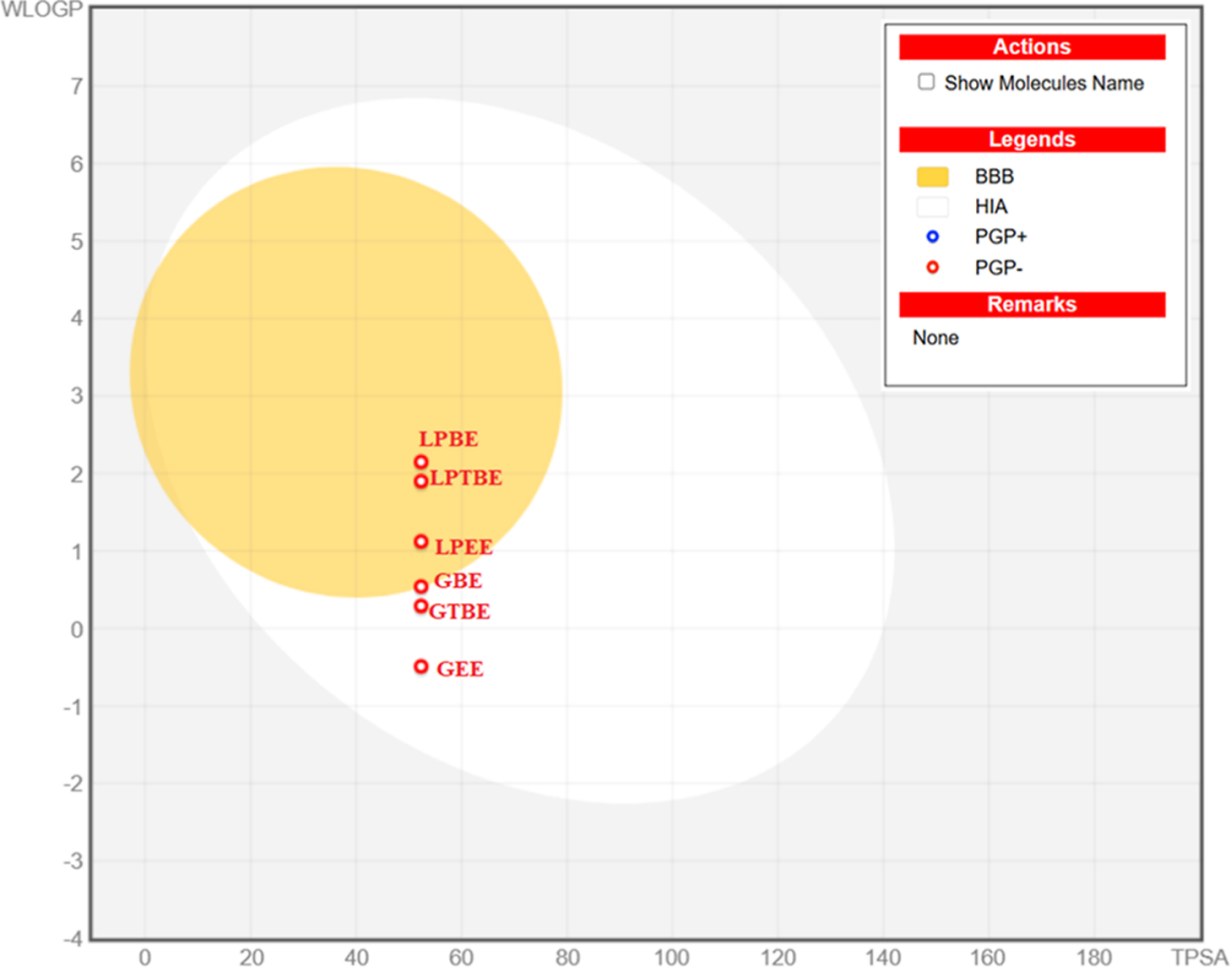
BOILED-EGG of all esters (GBE: glycine benzyl ester; GTBE/glycine *t*-butyl ester, GEE/glycine ethyl ester, LPBE:l-phenylalanine benzyl ester; LPTBE: l-phenylalanine *t*-butyl ester; and LPEE:l-phenylalanine ethyl ester).

**Figure 8:**
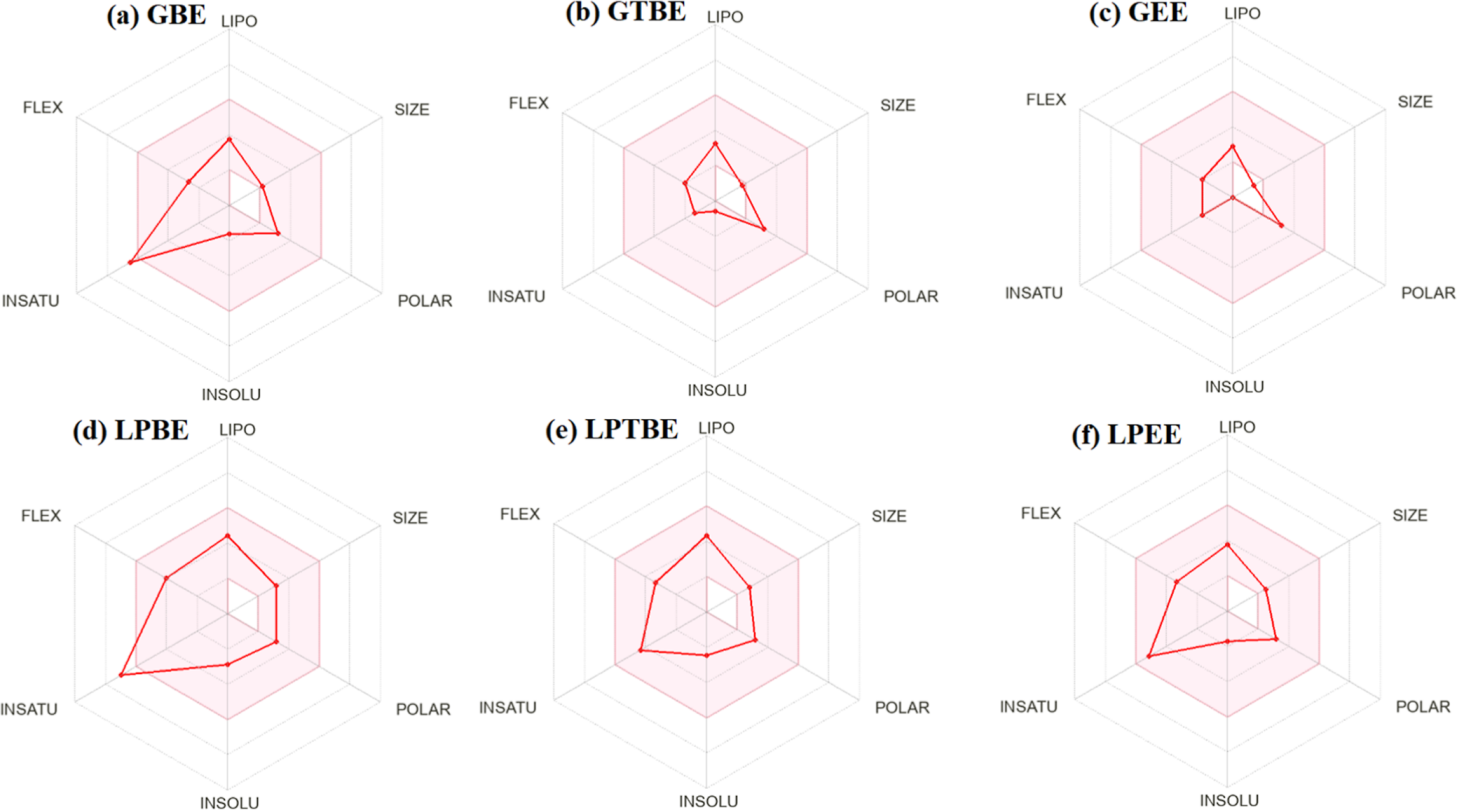
Bioavailability radar of the amino acid esters: (a) glycine benzyl ester, (b) glycine *t*-butyl ester, (c) glycine ethyl ester, (d) l-phenylalanine benzyl ester, (e) l-phenylalanine *t*-butyl ester, and (f) l-phenylalanine ethyl ester.

According to the BOILED-Egg model.

- GI Absorption: All molecules are predicted to have high gastrointestinal absorption.
- BBB Permeation: A clear distinction is observed based on molecular structure; while GEE and GTBE do not cross the blood–brain barrier, the more lipophilic derivatives (GBE, LPBE, LPTBE, and LPEE) are predicted to be BBB permeant, as indicated by their position in the ″yolk″ region.
- Efflux Mechanism: None of the compounds are substrates for *P*-glycoprotein (*P*-gp), suggesting they are not subject to active efflux and can maintain effective intracellular concentrations.

## 4 Discussion

This study aimed to evaluate the antimicrobial and antibiofilm activities of selected amino acid esters and to support the experimental findings through molecular docking and ADME analyses. According to broth dilution test results of the study, the antimicrobial activities of glycine esters against both *E. coli* and *S.aureus* ranked according to their alkyl groups are as follows: glycine benzyl > glycine *t*-butyl > glycine ethyl. Similarly, the antimicrobial activities of l-phenylalanine esters against both *E. coli* and *S.aureus* are as follows: l-phenylalanine benzyl > l-phenylalanine *t*-butyl > l-phenylalanine ethyl (Table 2). Disc diffusion test results also showed that the largest inhibition zones were observed with l-phenylalanine benzyl ester for both of the bacteria. The inhibitory activity of amino acid esters, particularly glycine benzyl, l-phenylalanine benzyl, and l-phenylalanine *t*-butyl ester, against *S. aureus* appears to be quite good. When overall results of inhibition efficiency against both bacteria are examined, l-phenylalanine benzyl ester is the most effective ester. Also, biofilm inhibition tests for *E. coli* and *S.aureus* showed that glycine esters were more effective than phenylalanine esters. The strong antibiofilm activity of glycine esters may indicate a mechanism independent of bactericidal effects and could be associated with interference in surface adhesion or the suppression of extracellular polymeric substance (EPS) production. The difference between liquid microdilution and disk diffusion results may be due to the limited diffusion of hydrophobic ester molecules in agar-based media and differences in the molecular size. Among the glycine esters, glycine benzyl ester showed the highest biofilm inhibition activity for *S. aureus*, while glycine ethyl ester showed the highest biofilm inhibition activity for *E. coli*. When MICs, MBCs, and ZOIs are evaluated together, it is seen that benzyl group esters exhibit a greater antimicrobial effect against both tested strains (Table 2 and Figure 1), and the antimicrobial effect caused by both esters follows this order: benzyl ester > *t*-butyl ester > ethyl ester. The benzyl ester group enhances hydrophobicity in both glycine and phenylalanine derivatives; however, this effect is limited in glycine derivatives (Table 2).

In general, in all esters, the conversion of the amino acid carboxyl group to the ester eliminated the negative charge, leading to a decrease in the overall polarity of the molecule. Increased lipophilicity facilitated passive diffusion across the hydrophobic layer of the bacterial membrane. Meanwhile, the conservation of the protonatable amino group ensured the continuation of strong electrostatic interactions, especially with negatively charged phospholipid head groups and lipopolysaccharides. The data obtained show that the activity varies depending on both the chemical structure of the amino acid side chain and the volumetric and lipophilic properties of the ester group. Benzyl and *tert*-butyl esters, compared to ethyl ester, provided increased lipophilicity and volumetric contribution, expanding the contact surface with hydrophobic residues and allowing for more stable complex formation.^41^ Specifically, it can be said that these esters interact more strongly with the bacterial cell membrane, disrupting the membrane integrity. These esters can be said to accumulate on the membrane surface, destabilizing the lipid bilayer and causing leakage of intracellular contents through a mechanism similar to the ″carpet model″. In the carpet mechanism, molecules bind parallel to the membrane surface and form a dense layer that destabilizes lipid packing in a detergent-like manner.^42^ Therefore, more hydrophobic ester derivatives are likely to exhibit antimicrobial activity mainly through mechanisms based on membrane interaction. In addition, the derivatives with sufficient hydrophobic character may penetrate into the membrane, trigger the formation of toroidal pores, and cause cell death by disrupting the ionic balance. In contrast, smaller and relatively more polar ethyl ester derivatives may cross the membrane and reach intracellular targets, exerting antibacterial effects through interactions with DNA/RNA or inhibition of protein synthesis. In this context, in Gram-negative bacteria, the presence of an outer membrane barrier enhances the effectiveness of more hydrophobic derivatives, whereas in Gram-positive bacteria, both membrane-disruptive and intracellular mechanisms may contribute. Overall, the findings suggest that the antibacterial effects of these compounds are not limited to a single mechanism but may involve multiple pathways, depending on their structural properties.

Studies in the literature support the role of structural parameters and the structure-activity relationship in determining the antimicrobial efficacy. Although there are a limited number of studies in the literature investigating the effectiveness of glycine and l-phenylalanine derivatives against different microorganisms, some studies on different amino acids are also available. These study results support our findings. Especially, recent studies on *N*-acyl glycine derivatives have shown that chain length, binding type, and functional group positioning significantly influence antimicrobial outcomes.^43^ At the same time, a study with a different ester of glycine determined that the activity of the compound depends on the balance of increasing lipophilicity and cellular penetration.^44^ In a recent study, it was stated that hydrophobic interaction and functional groups are important in the antibacterial properties of l-cysteine esters against *E. coli* and *S. aureus*.^45^ In another study using the sulfonamide phenylalanine analog series, the compounds were investigated against *E. coli* and *S. aureus*, and it was stated that the flexibility of the ring structure and the hydrophobic character of the molecule and the position and orientation of the functional group affect the biological activities.^46^ Similarly, research results on dihydroxyphenylalanine esters are also noteworthy. These esters have shown moderate antibacterial activity against both Gram-positive and Gram-negative bacterial species. The difference in the activity of the esters has been attributed to increased hydrophobic interactions between the surfactant tail and the bacterial cell.^47^ Also, in a different study, the importance of the aromatic ring was highlighted, and it was stated that the addition of l-phenylalanine to the antimicrobial peptide protonectin sequence enabled phenylalanine-prt to show significant selectivity against Gram-positive bacteria.^48^ These results specifically confirm the effect of functional-group-derived differences in esters on activity. The fact that the amino acid ester structure is more hydrophobic and contains an aromatic ring increases its biological activity.^49^ For example, curcumin is known to have various biological activities due to its antioxidant mechanism, and this aromatic polyphenol has been shown to be effective against *S. aureus* and *E. coli* due to its hydrophobic structure.^50^ Also, it has been reported in the literature that some D amino acids (leucine, methionine, tyrosine, tryptophan, proline and phenylalanine) exhibit antibacterial activity against *S. aureus* and *E. coli* and that amino acids inhibit bacterial biofilm formation by reducing cellular hydrophobicity and extracellular polysaccharide content.^51–53^ A 2026 study also showed that benzyloxycarbonylglycine effectively inhibits biofilm formation and disperses mature biofilms in both methicillin-susceptible and methicillin-resistant *S. aureus* strains, with antibacterial activity associated with bacterial membrane depolarization and increased membrane permeability.^54^

However, the functional groups of esters (-benzyl, -*t*-butyl, and -ethyl) cause small differences in the alkyl chain structure among similar molecules, while significantly affecting the acidity or basicity of the structure. According to the literature supporting this finding, the acid–base equilibrium constants of different amino acid esters, including glycine and l-phenylalanine, have been determined, and it has been stated that alkyl groups significantly affect the acid–base equilibrium, leading to differences in *pK*_*a*_ values.^55^ The obtained MIC/MBC results also reflect the effects of these differences in esters.

Furthermore, all studied amino acid esters exhibit favorable drug-like characteristics, adhering to Lipinski’s Rule of Five^56^ with zero violations, indicating their potential as viable drug candidates (Table S1). Lipinski’s Five Rules are considered one of the most fundamental filters in predicting drug-like properties, particularly oral bioavailability. According to these rules, parameters such as molecular weight, lipophilicity (logP), and hydrogen bond donor/acceptor number directly determine a compound’s ability to cross biological membranes and thus its ADME profile. Also, according to the BOILED-Egg model (Figure 7), the esters demonstrated a high probability of efficient absorption. Simultaneously, the bioavailability radar (Figure 8) confirms that the majority of these molecules fall within the optimal physicochemical space for lipophilicity, size, polarity, and solubility. While smaller derivatives like glycine ethyl ester show minor deviations in molecular weight and molar refractivity, all compounds maintain a consistent bioavailability score of 0.55, suggesting high oral drug-likeness. Furthermore, low synthetic accessibility scores (ranging from 1.00 to 2.42) indicate that these molecules are relatively easy to synthesize. The metabolic study indicates a low risk of drug–drug interactions, as most molecules do not inhibit major cyclochrome P450 (CYP) isoforms. However, the larger derivatives, l-phenylalanine benzyl ester and l-phenylalanine ethyl ester, are predicted as potential inhibitors of CYP2D6. Regarding excretion, all molecules exhibit “soluble” to “very soluble” water solubility profiles across various models (ESOL, Ali, and SILICOS-IT), which supports favorable pharmacokinetics and systemic distribution. Therefore, based on the results obtained, it can be said that degradation, membrane permeability, solubility, ester hydrolysis/stability, or possible efflux mechanisms differ among esters.^56–58^

Moreover, if we make a general assessment together with the docking analysis results, in this study, hydrogen bond interactions can be considered a key stabilizing force, particularly supporting the activity of phenylalanine derivatives. They stabilize the ligand position and contribute to the strong inhibitory effect by lowering the binding energy. The phenylalanine backbone enhances π–π stacking and hydrophobic interactions within the receptor’s binding pocket, promoting a more stable and energetically favorable ligand–protein complex. These interactions are primarily supported by the aromatic ring system of phenylalanine, which aligns with the hydrophobic residues in the active site, and by hydrogen bonds involving residues such as GLY209 and MET207, which provide additional structural stabilization.^59^ This structural complementarity between the ligand and the binding site is likely the reason for the experimentally observed antibacterial performance for l-phenylalanine benzyl and *t*-butyl ester, which exhibited the lowest MIC and MBC values among all compounds tested.

In contrast, the glycine esters without aromatic side chains (glycine *t*-butyl and ethyl ester) yielded weaker binding energies (−6.9 kcal/mol) and displayed higher MIC and MBC values, reflecting lower antibacterial potency. Conversely, glycine benzyl ester displayed moderate binding energy (−8.2 kcal/mol) yet produced lower MIC values compared with the penicillin–streptomycin control, suggesting that some glycine derivatives may act through alternative mechanisms such as biofilm disruption or membrane interference rather than direct enzyme inhibition.

These interactions support the observed experimental trends but should not be interpreted as definitive evidence of an antibacterial mechanism. In general, it can be said that for glycine derivatives as well, the contribution of the alkyl group in the structure to volume and lipophilicity affects activity, but aromatic benzyl substitution provided the most significant improvement. Overall, optimum activity appears to depend on a balanced combination of lipophilicity and aromatic side chain. Considering the obtained biological and docking and ADME data, the l-phenylalanine benzyl ester derivative appears to be a promising candidate, particularly in terms of its drug-like properties and potential for significant binding to the target protein.

## 5 Conclusion

The antimicrobial activities of the studied esters were influenced by the functional groups in the ester structure and the hydrophobic, aromatic, and amphipathic natures of the structure. The study results demonstrate that these findings indicate that structural complexity and aromaticity in phenylalanine ester derivatives contribute to stronger ligand–receptor stabilization compared with simpler glycine esters. Overall, the parallel between computational and experimental outcomes supports the predictive value of the docking model and highlights the l-phenylalanine benzyl ester as the most promising antibacterial candidate for further optimization and mechanistic exploration. Collectively, the synergy between computational predictions and experimental outcomes in this study provides a robust foundation for the rational design of next-generation antibacterial agents. Incorporating docking analysis for the control antibiotic in subsequent studies would elucidate the relative binding efficacy of these compounds and confirm their competitive or enhanced inhibitory capacity in comparison to traditional pharmaceuticals. No research has yet been conducted investigating the antimicrobial and antibiofilm effects of these amino acid ester forms of *E. coli* and *S. aureus* strains. Therefore, the results of this study are valuable. The results of this study will also contribute to the development of new synthesizable amino acid–based drugs.

## Supplementary Material



## Notes

3D shape of GBE+1HNJ; 3D shape of GTBE+1HNJ; 3D shape of LPEE+1HNJ; 3D shape of LPTBE+1HNJ; 3D shape of GEE+1HNJ; comparative ADME and physicochemical properties of amino acid esters (PDF)
