# Supplementary material for: Biological Evaluation of Some Amino Acids Esters: A Study on Antimicrobial, Antibiofilm, and Molecular Docking
Source: ACS Omega. 2026 Apr 4;11(15):23200–10. doi: 10.1021/acsomega.5c13628 (PMC13103763; doi:10.1021/acsomega.5c13628)
Supplement: Supplementary file 1 [file ao5c13628_si_001.pdf]

Supporting Informations

Biological Evaluation of Some Amino Acids Esters: A Study on Antimicrobial, Antibiofilm, and Molecular Docking

Tuğçe Deniz Karaca[1*]

[1]Gazi University, Vocational School of Health Services, Department of Medical Services and Techniques, 06830- Ankara, Türkiye

[*]Correspondence E-mail: tdenizkaraca@gazi.edu.tr

Figure S1. The 3D shape of GBE+1HNJ.

Figure S2. The 3D shape of GTBE+1HNJ.

Figure S3. The 3D shape of LPEE+1HNJ.

Figure S4. The 3D shape of LPTBE+1HNJ.

Figure S5. The 3D shape of GEE+1HNJ.

Table S1. Comparative ADME and Physicochemical Properties of Amino Acid Esters

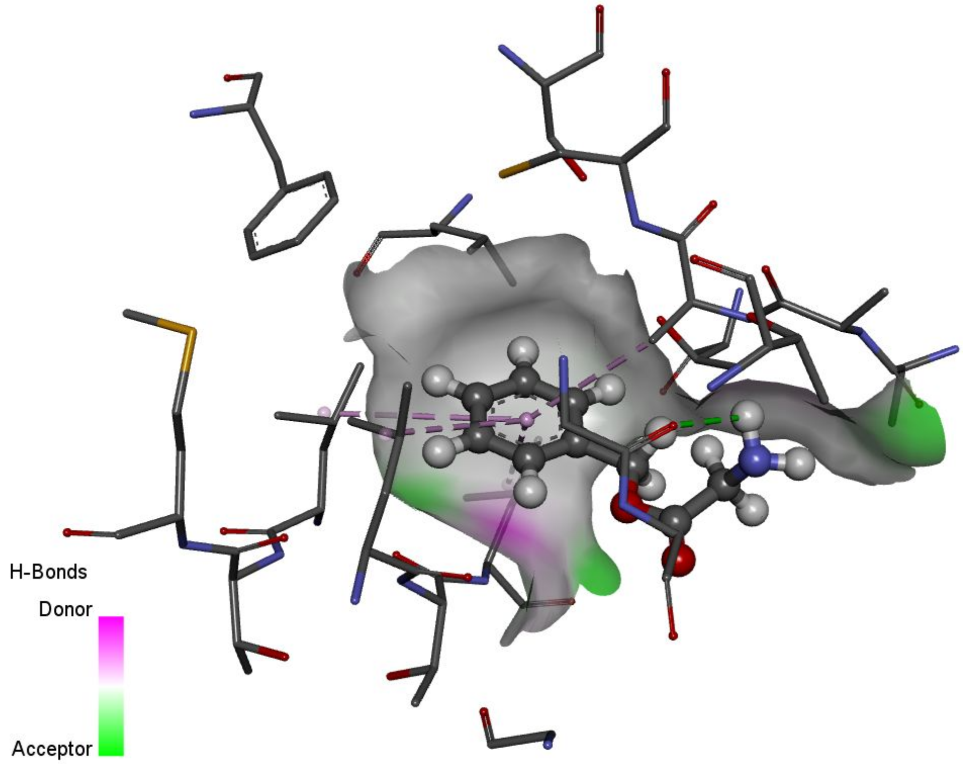

Figure S1. The 3D shape of GBE+1HNJ.

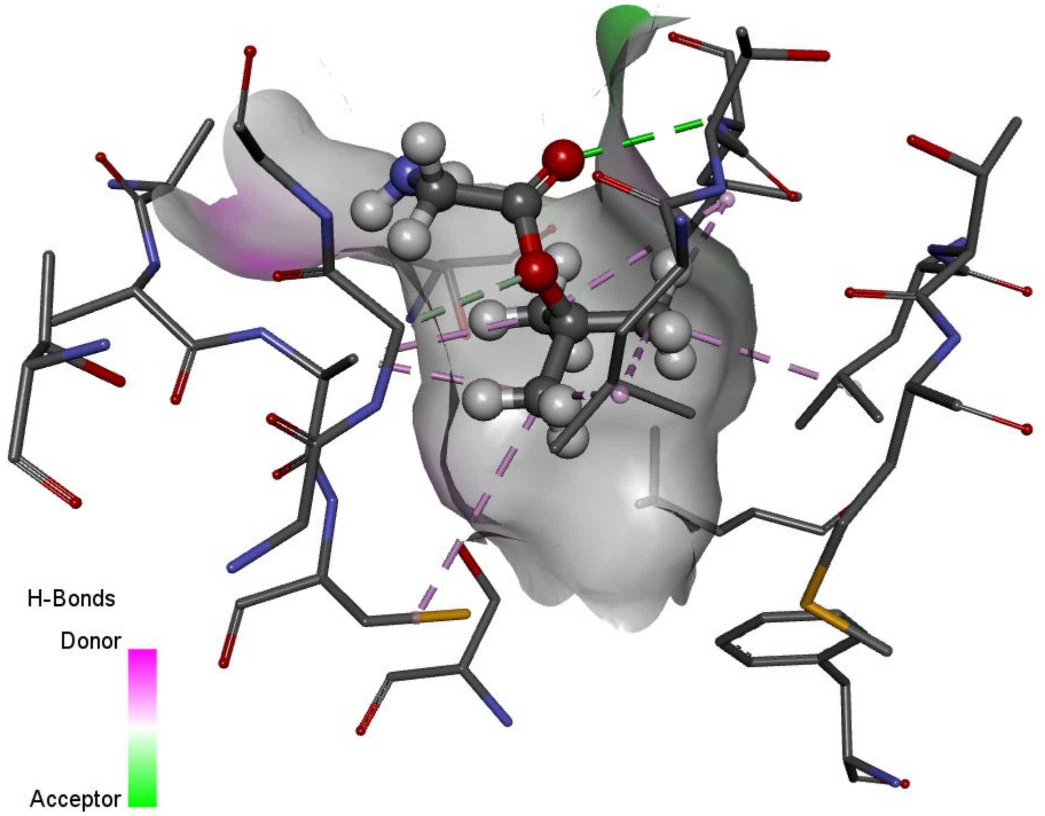

Figure S2. The 3D shape of GTBE+1HNJ

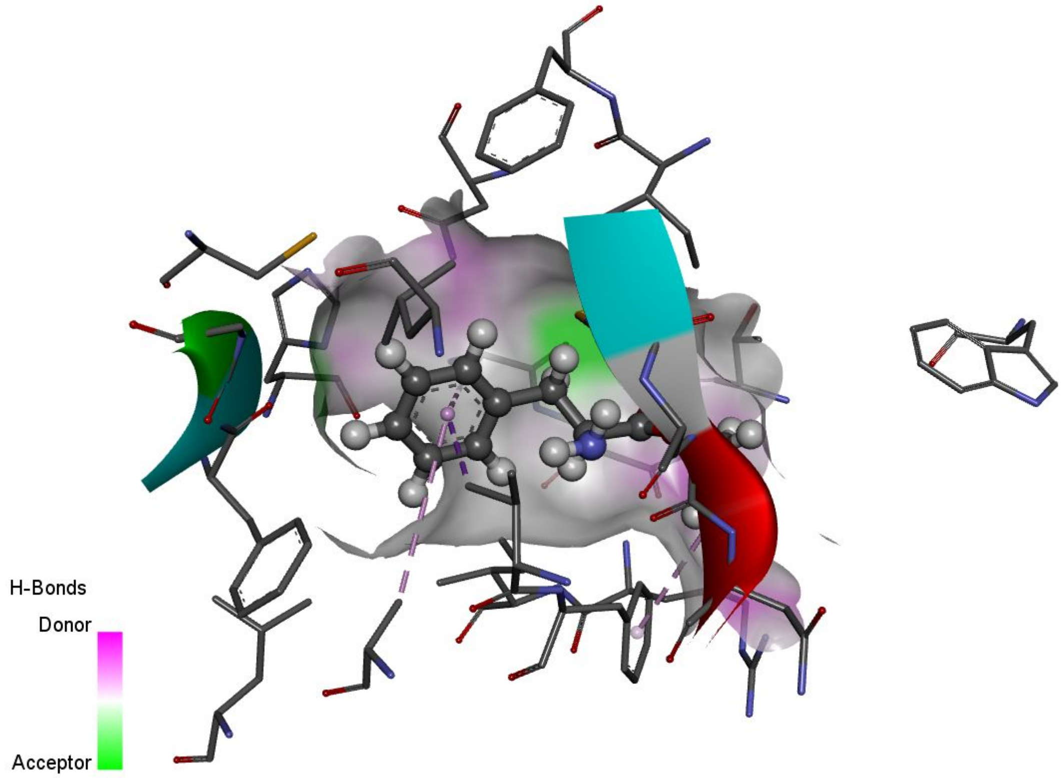

Figure S3. The 3D shape of LPEE+1HNJ.

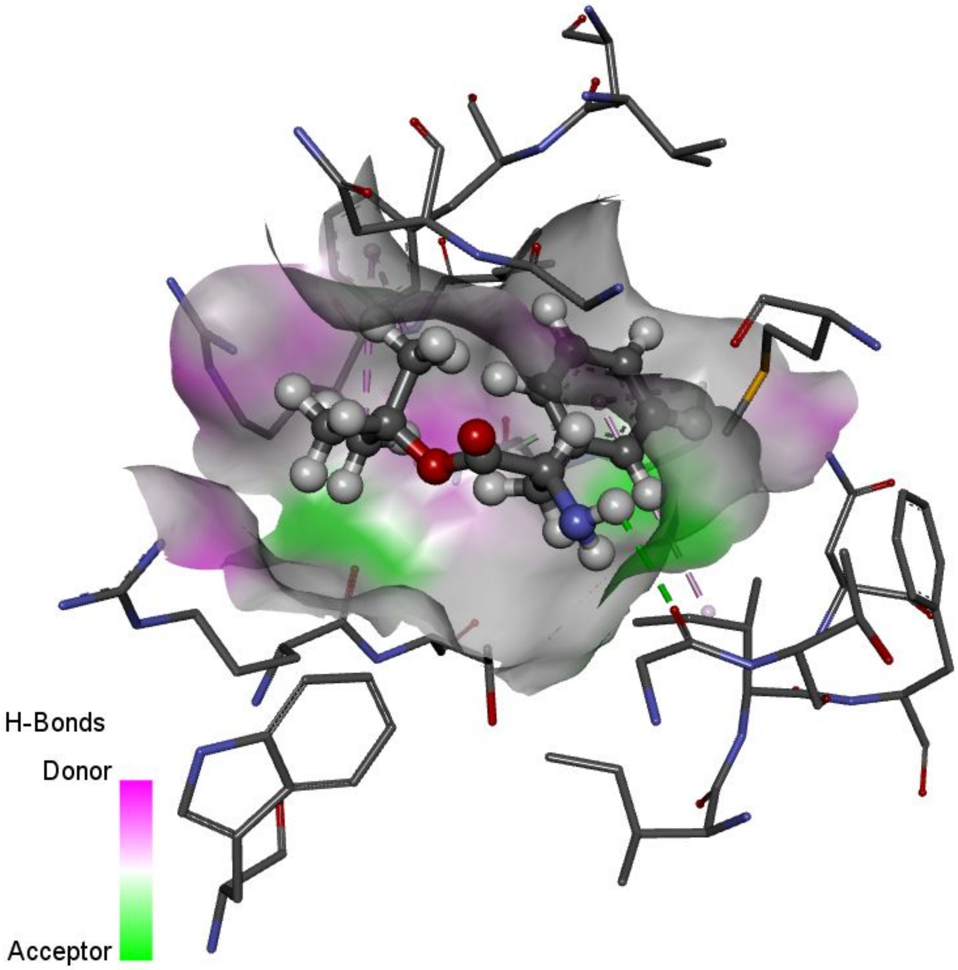

Figure S4. The 3D shape of LPTBE+1HNJ.

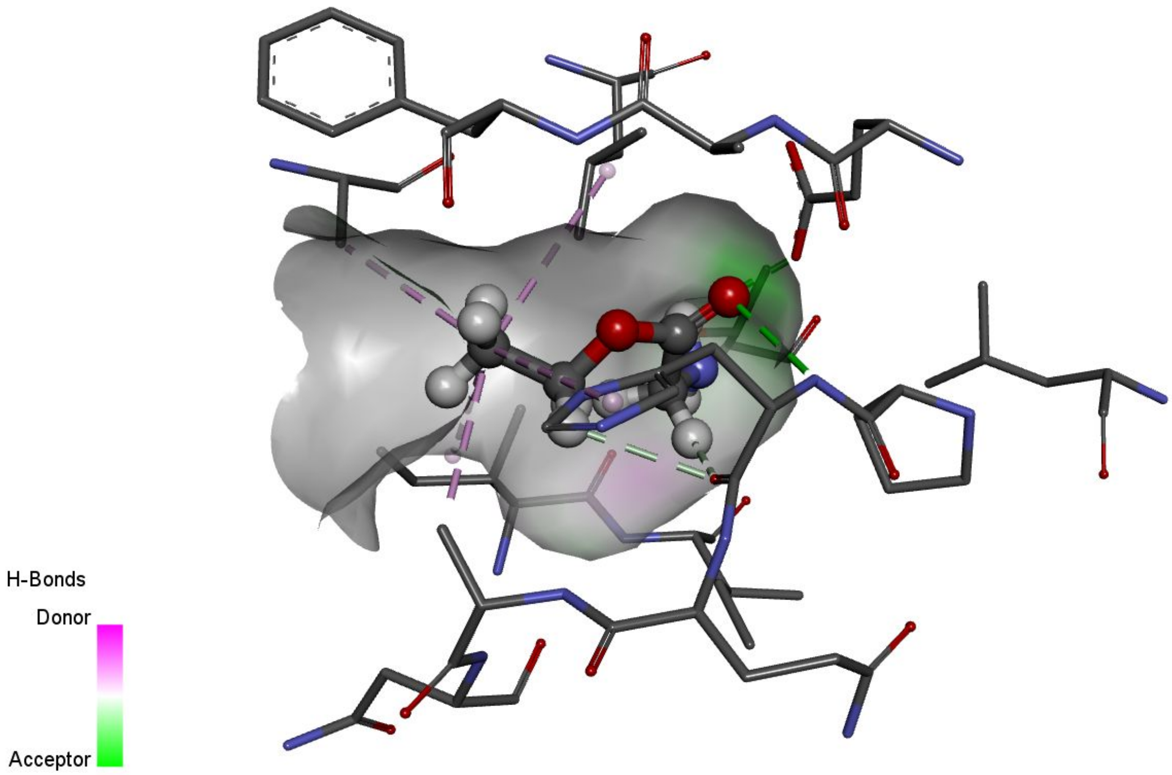

Figure S5. The 3D shape of GEE+1HNJ.

**Table S1.** Comparative ADME and Physicochemical Properties of Amino Acid Esters						
**Parameter**	**LPTBE**	**GEE**	**LPEE**	**LPBE**	**GTBE**	**GBE**
**Physicochemical Properties**						
Molecular Formula	$C_{13}H_{19}NO_2$	$C_4H_9NO_2$	$C_{11}H_{15}NO_2$	$C_{16}H_{17}NO_2$	$C_6H_{13}NO_2$	$C_9H_{11}NO_2$
Molecular Weight (g/mol)	221.30	103.12	193.24	255.31	131.17	165.19
Heavy atoms	16	7	14	19	9	12
Aromatic heavy atoms	6	0	6	12	0	6
Fraction Csp3	0.46	0.75	0.36	0.19	0.83	0.22
Rotatable bonds	5	3	5	6	3	4
H-bond acceptors	3	3	3	3	3	3
H-bond donors	1	1	1	1	1	1
Molar Refractivity	64.28	25.33	54.63	74.31	34.99	45.01
TPSA ($Å^2$)	52.32	52.32	52.32	52.32	52.32	52.32
**Lipophilicity, water solubility and Pharmacokinetics**						
Consensus LogP	2.13	-0.10	1.53	2.50	0.51	1.11
LogS (ESOL)	-2.46	-0.01	-1.71	-2.89	-0.57	-1.63
GI absorption	High	High	High	High	High	High
BBB permeant	Yes	No	Yes	Yes	No	Yes
P-gp substrate	No	No	No	No	No	No
CYP1A2 inhibitor	No	No	No	No	No	No
CYP2C19 inhibitor	No	No	No	No	No	No
CYP2C9 inhibitor	No	No	No	No	No	No
CYP2D6 inhibitor	No	No	No	Yes	No	No
CYP3A4 inhibitor	No	No	No	No	No	No
Log Kp (skin permeation)	-6.18	-7.23	-6.71	-6.29	-6.97	-6.56
**Druglikeness and Medicinal Chemistry**						
Lipinski rule	Yes (0 violation)	Yes (0 violation)	Yes (0 violation)	Yes (0 violation)	Yes (0 violation)	Yes (0 violation)
Bioavailability score	0.55	0.55	0.55	0.55	0.55	0.55
PAINS alert	0	0	0	0	0	0
Brenk alert	0	0	0	0	0	0
Synthetic accessibility	2.13	1.00	1.93	2.42	1.03	1.32
